# Patent applications for using DNA technologies to authenticate medicinal herbal material

**DOI:** 10.1186/1749-8546-4-21

**Published:** 2009-11-24

**Authors:** Pang-Chui Shaw, Ka-Lok Wong, Albert Wai-Kit Chan, Wai-Cheong Wong, Paul Pui-Hay But

**Affiliations:** 1 Department of Biochemistry, Chinese University of Hong Kong, Shatin, Hong Kong, PR China; 2 Institute of Chinese Medicine, Chinese University of Hong Kong, Shatin, Hong Kong, PR China; 3 Law Offices of Albert Wai-Kit Chan, PLLC, Whitestone, New York 11357, USA; 4 Department of Biology, Chinese University of Hong Kong, Shatin, Hong Kong, PR China

## Abstract

Herbal medicines are used in many countries for maintaining health and treating diseases. Their efficacy depends on the use of the correct materials, and life-threatening poisoning may occur if toxic adulterants or substitutes are administered instead. Identification of a medicinal material at the DNA level provides an objective and powerful tool for quality control. Extraction of high-quality DNA is the first crucial step in DNA authentication, followed by a battery of DNA techniques including whole genome fingerprinting, DNA sequencing and DNA microarray to establish the identity of the material. New or improved technologies have been developed and valuable data have been collected and compiled for DNA authentication. Some of these technologies and data are patentable. This article provides an overview of some recent patents that cover the extraction of DNA from medicinal materials, the amplification of DNA using improved reaction conditions, the generation of DNA sequences and fingerprints, and the development of high-throughput authentication methods. It also briefly explains why these patents have been granted.

## Background

Herbal medicines are used in many countries; unlike chemical drugs, herbal preparations often consist of a combination of materials. Life-threatening poisoning may occur if toxic adulterants or substitutes are used instead. In 1989, two individuals in Hong Kong suffered serious neuropathy and encephalopathy after consuming a broth made with the roots of *Podophyllum hexandrum *(*Taoerqi*), a toxic herb mistaken as *Gentiana rigescens *(*Dianlongdan*) [1]. In 2001, 63 people in the Netherlands reported symptoms of general malaise, nausea, and vomiting 2 to 4 hours following consumption of an herbal tea containing *Illicium anisatum *(Japanese star anise) [2]. Aristolochic acid nephropathy has also been reported in Hong Kong [3], Korea [4], and Belgium [5] as a result of herbs contaminated with aristolochic acids.

Herbal medicinal products of high quality are essential for consumer confidence. Many countries and regions have set up quality standard tests for imported herbal medicine. For example, 324 Chinese medicinal materials are regulated by the Department of Health in Taiwan [6]. Herbal manufacturers must label their product with the names of the herbs in the product, and products without such labels are prohibited from entering Taiwan.

Herbal medicinal materials are traditionally identified by their organoleptic or microscopic characteristics, including size, shape, color, odor, flavor, texture and other physical properties. Although the methods based on these characteristics are simple, their accuracy strongly depends on the experience of the inspector, who may or may not be aware of the subtle differences in the structures among related species. Chemical analysis is powerful but the results are affected by the physiological and storage conditions of the herbs. Closely related species containing similar chemical components may also confuse the identification.

DNA technology provides a useful and independent tool to complement chemical analyses for the authentication and quality assurance of medicinal materials. DNA technology offers four advantages: (1) DNA-based markers are less affected by age and physiological conditions; (2) any part of the herb can be collected for analysis; (3) only a small sample is necessary for analysis; and (4) some DNA regions may be species-specific, whereas others may be family-specific.

The principles and techniques of DNA methods were recently reviewed [7,8]. Therefore, only a brief account of the popular techniques for DNA authentication is given as an introduction.

A major approach in DNA authentication is whole-genome fingerprinting. The common methods include random amplified polymorphic DNA (RAPD) [9], simple sequence repeat (SSR) [10], amplified fragment length polymorphism (AFLP) [11], and direct amplification of length polymorphism (DALP) [12]. These methods do not require any prior knowledge of the target DNA sequence and, in general, allow for quick identification of genetic polymorphism [13-16].

Owing to the processing procedures, DNA of herbal material is usually somewhat degraded. For accurate DNA fingerprints, attention must be paid to certain defined regions of the genome. Approaches available include polymerase chain reaction-restriction fragment length polymorphism (PCR-RFLP) [17-19] and sequence characterized amplified region (SCAR). The latter involves first sequencing the polymorphic bands from the whole-genome fingerprinting and then using them as reference markers. Multiplex PCR may be employed to examine several SCAR markers simultaneously [20,21].

DNA sequencing is the most definitive means for revealing the identity of an unknown sample [22]. For high-throughput authentication, DNA microarrays may be used. The steps include designing specific probes for a species, fabricating them onto a support, hybridizing with fluorescent-labeled fragments amplified from the genomic DNA of the herbal mixture and reading the hybridization signals with a scanner. A DNA microarray has been established for the identification of a *Dendrobium *species from a medicinal formula containing nine herbal components [23] and for the differentiation of *Dendrobium officinale *from other *Dendrobium *species [24].

In this article, we first outline the procedures of patent application for readers who are not familiar with the process. Then, we describe the existing patents for using DNA methods to authenticate medicinal materials and illustrate how they overcome the limitations of the existing technologies. We also evaluate their market potential and suggest ways to improve them. We hope readers will appreciate how existing DNA technologies may be applied in novel ways for the authentication of medicinal materials as well as opportunities for patent application.

### Patents and the application process

For a United States (US) patent to be issued, an invention must be novel, non-obvious, and useful [25]. Section 101 of Title 35 of the US Codes (35 USC §101) states that " [w]hoever invents or discovers any new and useful process, machine, manufacture, or composition of matter, or any new and useful improvement thereof, may obtain a patent therefore, subject to the conditions and requirements of this title." Furthermore, " [a] patent may not be obtained though the invention that is not identically disclosed or described ..., if the differences between the subject matter sought to be patented and the prior art are such that the subject matter as a whole would have been obvious at the time the invention was made to a person having ordinary skill in the art to which said subject matter pertains" (35 USC §103). The patentee is granted the right to exclude others from making, using, offering to sell or importing the invention into the US for a period of 20 years from the date of filing the patent application. There are three types of patents in the US, namely utility, design, and plant patents. Patents for biotechnology are mostly utility patents.

**Figure 1:**
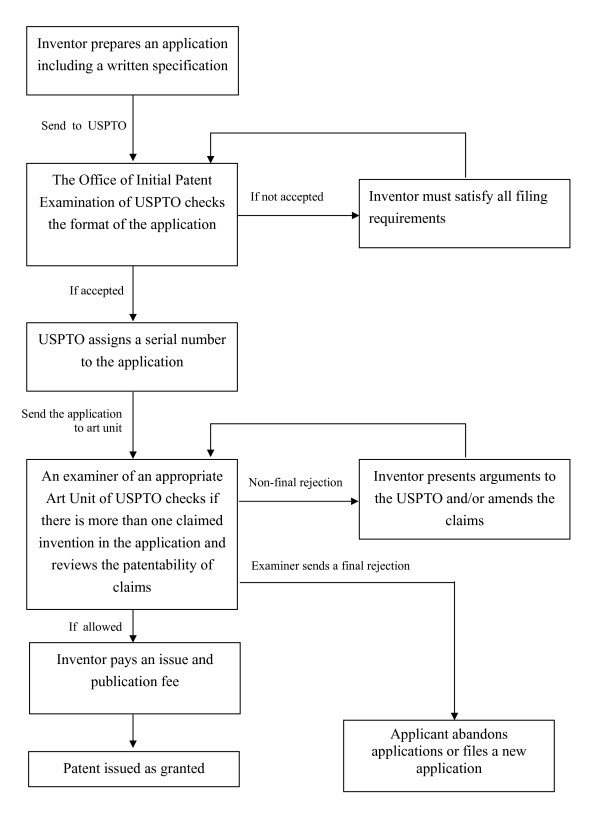
**Flow chart for US patent application **[25].

The process starts with filing an application with the US Patent and Trademark Office (USPTO) (Figure 1). The application should include a written description (also known as specification) of the invention and of the manner and process of making and using it in full, clear, concise and exact terms to enable a person skilled in the art to make and use the invention. In addition, the application must describe the best mode considered by the inventor of carrying out the invention. The application should also include at least one claim setting forth the metes and bounds of the subject matter to be protected. The Office of Initial Patent Examination of the USPTO reviews the application to see if all initial requirements have been met. The application is then assigned a serial number, and an examiner from the relevant art unit of the USPTO is assigned to assess the application [26]. The examiner will assess the claims on their merits. If objections or rejections to some or all of the claims are raised, the applicant will be given an opportunity to amend the claims and/or refute the objections or rejections, until the examiner is convinced that the claims are allowable. After receiving a notice of allowance and paying an issue fee, the applicant will receive a patent on the invention as defined by the allowed claims.

A Chinese patent may be obtained by applying to the State Intellectual Property Office (SIPO) of the People's Republic of China [27]. Unlike in the US where the first person who invents is honored, in China's system the first person who invents and files an application for a patent is entitled to a patent. In the US, an inventor has one year from the date of the first public disclosure of an invention to file an application for patent protection, whereas China does not usually allow such a grace period. Apart from these differences, the US and China have similar criteria for patentability regarding what is known as a utility patent. The Chinese Guidelines for Examination issued in 2006 by the SIPO uses the term "inventive step" instead of "non-obviousness" for judging the patentability. In Article 22.3 of the guidelines, " [i]nventive step of invention means that, as compared with the existing technology before the date of filing, the invention has prominent substantive features and represents notable progress." An invention is said to have prominent substantive features if, "having regard to the prior art, it is non-obvious to a person skilled in the art" (Guidelines for Examination). In other words, " [i]f the person skilled in the art can obtain the invention just by logical analysis, inference or limited experimentation on the basis of the prior art, the invention is obvious and therefore has no prominent substantive features" (Guidelines for Examination).

A utility patent in the US is equivalent to an invention patent in China. However, like many other countries, China has what are known as utility model patents, which are reserved for "any new technical solution relating to the shape, the structure, or their combination, of a product, which is fit for practical use" (Guidelines for Examination). Although a utility model patent is usually issued much faster than an invention patent because of the less stringent examination process, protection from a utility model patent is only good for 10 years as compared to 20 years for an invention patent. The inventions described later in this article probably cannot be protected by a utility model patent except for the matrix mill under US Patent 6063616.

To obtain patent protection in multiple countries, inventors may file an international application under the Patent Cooperation Treaty. The United States and China are among the approximately 139 contracting countries of the treaty. After filing an international application, the applicant has a period of typically 30 to 31 months following the priority date (usually the filing date of a prior application that the international application claims priority of) to file national stage applications in any of the contracting countries. An international search report and written opinion are normally issued by an international searching authority for the international application prior to the timeline to enter a national stage. These reports cite any prior art and give an initial evaluation on patentability of the claims, so the applicant would have an opportunity to determine if and where he or she wants to seek protection of the invention. If applications are then submitted to individual countries, they will be examined based on the international written opinion and according to local patent laws and rules. Any patent later issued will be enforceable only within the country of issue.

Since the 1990s, DNA techniques have become popular for the authentication and quality control of Chinese medicinal material. For the protection of intellectual properties and opportunities for commercialization, patents have been applied for the extraction of DNA from medicinal material, amplification of DNA from difficult templates, generation of species-specific fingerprints and sequences, and high-throughput detection and identification of unknown DNA. In this article, recent US and Chinese patents relating to the extraction and amplification of DNA and the generation of DNA fingerprints and sequences for Chinese medicinal materials are described as examples for explaining why some of these patents have been allowed (Additional file 1) while other applications were abandoned (Additional file 2) so that the readers can appreciate the criteria.

### Patents for extraction of nucleic acids

**Figure 2:**
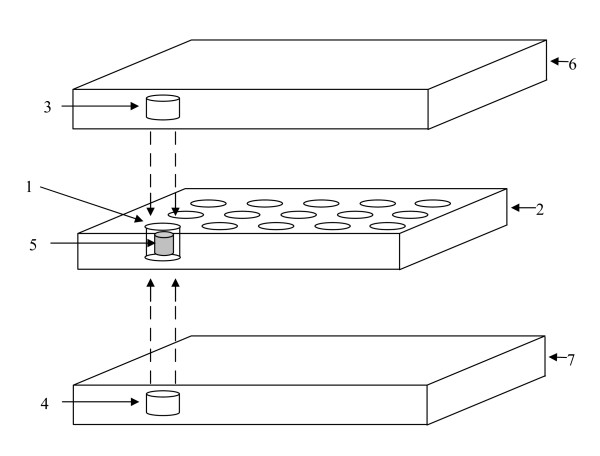
**Drawing and operation of the matrix mill for DNA extraction (adopted from US Patent 6063616)**. Sample is first added into the well (1) of the assay plate (2). The assay plate is then placed into the matrix mill with upper and lower magnetic core and coil assemblies (3, 4) in proximity. The grinding rod (5) is inserted into the well (1). Once alternating current passes through the upper coil assemblies (3, 4) in the frames (6, 7), the magnetically active grinding rod (5) begins the maceration of the sample material within the well.

The first step in DNA extraction is to grind or cut the sample into small pieces. Traditionally, this has been achieved by methods that use chemical, sonic wave, mechanical, or physical pressure systems. Sometimes hazardous liquid nitrogen or mortar and pestle are used. As such, these methods are often expensive, require too much effort for large-scale extractions, and provide low throughput. To accelerate and simplify the extraction process, Weeden *et al. *invented an innovative matrix mill (US Patent 6063616) that can isolate genomic DNA from numerous samples simultaneously [28]. Samples are added into a 96-well plate and a micropestle composed of a ferrous core coated with a non-reactive material being inserted into each tube (Figure 2). The plate is then placed onto the matrix mill, which provides electromagnetic energy for the movement of the pestle inside the tubes and acts as the random motion of a manual mortar and pestle. Studies showed that this matrix mill gave excellent DNA extracts for tissue types including pea, bean, cucumber, pepper and broccoli. Up to 96 samples can be prepared in each run, and the quality of DNA is sufficient for PCR. The authors believe that adding a chilling device to the matrix mill would be beneficial because the sample can then be processed under low temperature to avoid the degradation of DNA and RNA samples. Reducing the size of the micropestle would expand the capacity of the mill to a 384-plate format.

DNA with a high molecular weight that is free from contaminants ensures the success of molecular authentication. Generally, lysis buffer for DNA extraction comprises buffering ions, surfactants, chelators and proteinase. Chelators such as EDTA can bind to the free divalent ions to inhibit the enzymatic activities of some nucleases. Proteinase K digests the tissue or cell membrane and increases the yield of extraction. However, this Ca^2+^-dependent enzyme may not have a significant effect because divalent ions in the buffer are tightly bound to the chelators. US Patent 7214484 was issued for a lysis buffer that does not require a high concentration of chelators, allowing proteinase K to effectively digest the protein molecules in a sample and resulting in an increase in the efficiency of cell lysis [29]. This method is more effective in extracting DNA from animal than herbal material, as the former has higher protein content than the latter. To improve the lysis buffer for DNA extraction, we propose modifying proteinase K to make it less ion-dependent or replacing proteinase K altogether with a proteinase that is ion-independent.

For DNA isolation, organic solvents such as phenol and chloroform are commonly used. After centrifugation, DNA stays in the upper aqueous phase, whereas protein contaminants are denatured and partitioned either with the organic phase or at the interface between the organic and aqueous phases. The aqueous phase containing DNA molecules is then aspirated. However, the aspirated aqueous phase is sometimes contaminated with organic solvent because the aqueous-organic interface is not very stable. Non-polar phenolic compounds of the herbal material may inhibit downstream reactions, such as PCR. US Patent 5106966 was issued for an innovative DNA extraction method using polyester silica gel in the extraction medium [30]. Before centrifugation, polyester silica gel is placed in the bottom of the tube. This gel moves and separates the aqueous phase from the organic phase after centrifugation, acting as a barrier to permit decanting of the aqueous solvent. This method produces 40% more DNA than the one without polyester silica gel. Moreover, the ratio of absorbance at 260-280 nm of the purified DNA is consistently 1.8, which means the purified preparation is free from protein contamination.

### Patents for the amplification of difficult templates

After extracting the genomic DNA of the medicinal material, the next step is to employ PCR to generate a DNA fingerprint or obtain a particular region for further study. PCR is an enzymatic reaction that can amplify a defined region of the template DNA from a tiny amount of source material. It has become an indispensable technique used in biological research and has broad applications in DNA cloning, DNA sequencing, molecular phylogenetics, and molecular diagnosis. The PCR patent was granted to Cetus Corporation in 1987 [31] and later sold to Roche Molecular Systems. Since the invention in 1984, many related patented techniques have been developed, such as directional cloning for inserting a gene in a correct frame [32], multiplex PCR for amplifying multiple regions of genomic DNA [33], reverse transcription PCR for amplifying a defined piece of RNA [34], and hot-start PCR for increasing the specificity of the reaction [35]. Some of these patented techniques have been adopted for the amplification and cloning of DNA from medicinal material.

PCR may produce a negative result due to the presence of inhibitors or the secondary structure of the template or primer. The secondary structure of primers reduces the efficiency or even inhibits the binding process. High GC content of the DNA template in some medicinal materials also increases the melting temperature and makes the amplification process difficult. Some researchers tried to solve the problem by increasing the denaturation temperature from the conventional 94°C to 98°C or extending the denaturation time. Nevertheless, DNA polymerase may lose its activity under prolonged elevated temperature conditions. As such, the reaction buffer must be modified to protect DNA polymerase from degradation. US Patent 7150980 was issued for the use of proline, 2-methyl-4-carboxy-5-hydroxy-3,4,5,6,-tetrahydropyrimidine THP(A) and 2-methyl-4-carboxy-3,4,5,6-tetrahydropyrimidine THP(B) to increase the thermal stability of DNA polymerases at high temperatures [36], whereas THP(B) can lower the melting temperature of double-stranded DNA. US Patent 6783940 was issued for a technique that further improves the PCR condition by including sorbitol or sorbitol and DMSO in the PCR mixture, which is effective for reducing the non-specific amplification [37]. Sorbitol at a concentration of 0.15 M results in an increase in specific target DNA.

Hot-start PCR can increase the reaction specificity by inhibiting the activity of DNA polymerase during the sample preparation step. In hot-start PCR, DNA polymerase is bound to an antibody that is inactivated and dissociated from the DNA polymerase by heat denaturation. The major drawback is that the anti-polymerase antibody is expensive and only specific to a particular polymerase. US Patent 6403341 was issued for a method of inactivating the DNA polymerase by keeping the concentration of magnesium ions low [38], and this method is as efficient as hot-start PCR. The magnesium ions added in the reaction mixture are in precipitate form. Without magnesium ions, the DNA polymerase is inactive and no non-specific reaction occurs. Upon raising the temperature, magnesium ions are released from the precipitate. Magnesium ions are mixed with various phosphates in a tube to form precipitate. After precipitation, PCR components are added into the tube and PCR is then performed. In our view, this method may not be easy to reproduce as it is difficult to aliquot the precipitate accurately into individual tubes. Researchers should find a form of sequestered magnesium ions that can be stored and aliquoted as a solution. Upon heating, the sequestered ions are then released and the PCR reaction takes place.

The amplification success rate of PCR can be further increased by finding a superior polymerase or a better formulated PCR buffer. Such improvements would enhance the DNA amplification processes and may decrease the amount of template required. The latter is relevant to the amplification of DNA from medicinal materials, as in many cases the DNA of processed material is difficult to extract or highly degraded. Also, DNA extracted from dried medicinal materials may be contaminated with phytochemicals that can inhibit the amplification process. Improvements in the PCR buffer and DNA polymerase may overcome such inhibition and increase the amplification success rate.

### Patents for the generation of species-specific fingerprints and sequences

Commercial ginseng products come in the form of powder or shredded slices, rendering authentication by morphological and histological methods unpractical. Chemical analysis is limited by the available amount of chemical markers, ginsenosides, which are significantly affected by such factors as storage conditions, freshness of products, and post-harvesting processing. The techniques described in this section are patentable because they are improved technologies that can solve the authentication difficulties.

US Patent 5876977 was issued for a method of fingerprinting ginseng by taking advantage of the unique PCR-RFLP patterns of different ginsengs [39]. The form or the physical and chemical conditions of the sample do not appear to affect the outcome of the method. The internal transcribed spacer (ITS) region of the rDNA of herbal material is highly polymorphic between different species and is amplified by primers that bind to the conserved regions. The PCR products are then fragmented by selected restriction endonuclease. After electrophoresis, discrete and species-specific RFLP patterns are generated. The ITS sequences of several medicinal species, including *Panax ginseng *(*Renshen*), *Panax quinquefolius (Xiyangshen)*, and *Codonopsis *(*Dangshen*) species, have been patented. More than one distinctive RFLP profiles can be generated by digesting these DNAs with different restriction enzymes, which makes the interpretation of results straightforward. Chinese Patent 01102434 employs the same approach to authenticate other herbal materials [40]. The ITS sequences of 16 medicinal *Dendrobium *species and adulterant *Pholidota cantonensi *may also be beneficial to the horticulture industry. Patents for authenticating high-value Chinese medicinal materials are summarized in Table 1.

**Table 1:** Patents for using DNA techniques to authenticate high-value Chinese medicinal materials

Medicinal material	Description of the patent	DNA techniques	Patent number
*Radix Et Rhizoma Ginseng *(*Renshen*)	Distinguish *Panax ginseng *from *Panax quinquefolius*, distinguish *Codonopsis *species and adulterants	PCR-RFLP	US Patent 5876977
	Differentiate wild ginseng, cultivated ginseng, and adulterants	PCR-RFLP	Chinese Patent 200410016240
	Distinguish *Panax ginseng *from *Panax quinquefolius *and adulterants	SCAR	US Patent 6803215
*Caulis Dendrobii *(*Shihu*)	Distinguish 16 *Dendrobium *species and adulterants from each other	PCR-RFLP	Chinese Patent 01102434
*Cordyceps *(*Dongchongxiacao*)	Distinguish *Cordyceps sinensis *from seven related species	PCR-RFLP	Chinese Patent 99106135
	Distinguish *Cordyceps sinensis *from seven related species	PCR-RFLP	US Patent 6271003
	Distinguish *Cordyceps sinensis *from seven related species	DNA sequencing	US Patent 6251606
*Colla Corii Asini* (*Ejiao*)	Distinguish *Equus asinus *from domestic animals	PCR-RFLP	Chinese Patent 03153838
*Rhizoma Gastrodiae* (*Tianma*)	Distinguish *Gastrodia eleata *from adulterant *Lycopus lucidus*	SCAR	Chinese Patent 200510031346

Besides the ITS region, 5S rRNA intergenic spacer (5S rRNA) has also been employed to authenticate medicinal material. US Patent 6569625 was issued for a method of differentiating four medicinal *Fritillaria *species using this DNA region [41]. 5S rRNA is amplified by PCR and then sequenced. Each *Fritillaria *species has a unique spacer sequence, with the highest similarity between *Fritillaria anhuiensis *and *Fritillaria puqiensis *(97.48%). After digestion with *Eco*RI, fragments of 384 bp and 314 bp from *Fritillaria cirrhosa *and 460 bp and 123 bp from *Fritillaria thunbergii *are obtained, while the PCR products of *Fritillaria anhuiensis *and *Fritillaria puqiensis *are not cleaved. When using PCR-RFLP for rapid screening of species, the amplified region should be chosen carefully. For example, 5S rRNA intergenic spacer is a highly variable region, but there are many point mutations, insertions, and deletions among different copies of the gene in some species. As a result, the restriction profiles may not be consistent. We think that looking at the ITS or chloroplast regions of *Fritillaria *species may be more beneficial.

PCR-RFLP has also been used to authenticate drugs from animal sources such as the skin of donkey (*Equus asinus *L.), which is the raw material for the production of *Ejiao *used for treating anemia with dizziness, palpitation, muscular weakness; excessive menstrual discharge and tremors due to blood deficiency [42]. It is difficult to distinguish between *Ejiao *made from donkey and that from cow or horse because protein compositions from these animals are similar; therefore, using mass spectrometry or enzymatic analysis would not be helpful. Chinese Patent 03153838 was issued for a method of distinguishing *Equus asinus *from other domestic animals by amplifying partial cytochrome b gene regions (about 359 bp) from the skin of donkey, horse, cow, and mule [43]. PCR-RFLP profiles are generated by digestion of DNA with *Hin*fI, *Hae*III, *Alu*I, *Mbo*I, *Taq*I, and *Mse*I. The resolution of this method could be increased if the DNA sequences of these animals were determined in order to provide more information.

Another group has designed a testing kit for distinguishing *Eriocheir japonica sinensis *(hairy crabs) from *Eriocheir japonica hepuensis*. Hairy crab is popular with the medicinal value of removing heat from the stomach and liver and healing bone fractures [44]. Chinese Patent 01127215 was issued for a testing kit with a pair of specific primers and *Dra*I restriction enzyme [45]. A 293-bp DNA fragment is amplified with the specific primers and then digested with *Dra*I. For *Eriocheir japonica hepuensis*, the DNA is cleaved at position 188 and two fragments are generated. There is no *Dra*I site in the corresponding DNA of *Eriocheir japonica sinensis*, and therefore its DNA remains intact after digestion.

*Cordyceps *is a composite consisting of the stroma of the fungus *Cordyceps sinensis*, which parasitizes on the caterpillars of some moth. It is used to tonify the lung and kidney, arrest bleeding, and dissolve phlegm. Despite the many adulterants in the herbal market, prior classification methods based on configuration, physiology, or biochemistry could not provide an accurate and reliable classification of *Cordyceps sinensis*. Chinese Patent 99106135 and US Patent 6271003 were issued for a novel method for distinguishing *Cordyceps sinensis *from seven closely related fungal species [46,47]. The 18S rRNA region of genomic DNA is amplified and the PCR products are digested separately with four restriction enzymes. Based on the generated fingerprints, *Cfo*I produces restriction fragments of 570, 300, and 50 bp in *C. sinensis*. *Phytocordyceps ninchukispora *also has three fragments, but the sizes are different (830, 320, and 150 bp). Five fragments are produced in *Cordyceps militaris*, and the PCR products of five other fungal species are not cleaved. The 18S rRNA gene was then sequenced and compared in US Patent 6251606 [48]. All *Cordyceps sinensis *specimens have the same 18S rRNA gene sequence, which distinguishes them from related species. As compared with PCR-RFLP, DNA sequencing provides more information down to the base level while PCR-RFLP is much faster and is more suitable for screening. While the technology gives clear PCR-RFLP fingerprints for the fungi, it may not be easy to apply to *Cordyceps*, because both the 18S rRNA sequence of *Cordyceps sinensis *and the moth are amplified, and the resulting PCR-RFLP pattern may be confusing. For improvement, it may be necessary to design fungal-specific primers so that only the fungal DNA is amplified. In addition, because the 18S rRNA gene is rather conserved, possibly leading to inadequate resolution, other highly variable regions, such as an ITS, should be considered.

**Figure 3:**
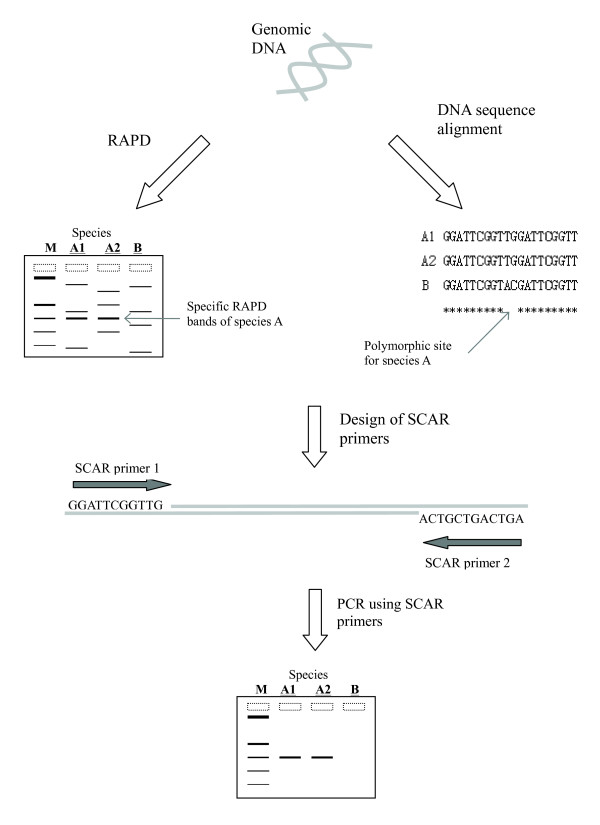
**SCAR primers can be designed from a whole-genome fingerprint, such as RAPD analysis or comparison of the DNA sequences among species**. During RAPD, genomic DNA is amplified by a RAPD primer. A polymorphic band is recovered from agarose gel and then sequenced. The RAPD band is turned into a species-specific SCAR marker.

Random amplified polymorphic DNA (RAPD) is another simple and fast DNA technique for generating fingerprints for medicinal materials. To identify an unknown sample, DNA from a known species is used as a standard control. After electrophoresis, the identity of the sample can be determined by matching it to the profile of a reference sample. However, RAPD fingerprints may be affected by the purity and integrity of the DNA template, type of DNA polymerase, and thermocycler used. On the other hand, polymorphic bands generated from RAPD are a good starting point for performing SCAR (Figure 3). US Patent 6803215 was issued for the use of SCAR to differentiate plant and animal medicinal materials, including *Panax *and medicinal snakes [49]. SCAR requires only one PCR step; thus, SCAR is more efficient than the PCR-RFLP approach described in US Patent 5876977 [39]. Polymorphic regions of ginseng were first identified by RAPD or direct amplification of length polymorphism (DALP), and several sets of SCAR primers were then designed to distinguish *Panax ginseng *and *Panax quinquefolius *and their adulterants. For animal samples, polymorphic bands that distinguished between the snake species *Agkistrodon actus*, *Bungarus multicinctus*, and *Zaocys dhumandes *were first produced by RAPD. These bands were sequenced for the design of SCAR primers, which are species-specific. The primers do not amplify DNA of other snake species or common domestic animals.

Chinese Patent 200410016240 was issued for a technique that differentiates wild ginseng from cultivated ginseng and adulterants. Two set of primers were designed based on the polymorphic DNA generated by DALP [50]. The first primer set only amplifies the genomic DNA of cultivated ginseng and produces a 174-bp PCR product. The second primer set can detect wild and cultivated ginseng and produces a 300-bp PCR product. These four primers can be used in multiplex PCR, which greatly increases the efficiency of the test.

SCAR primers can also be designed from an internal polymorphic site of the DNA (Figure 3). Chinese Patent 00134133 was issued for a process of generating SCAR primers using this approach [51]. *Radix et Rhizoma Rhei *(*Dahuang*) is the dried root and rhizome of *Rheum palmatum*, *R. tanguticum*, or *R. officinale*. Its indications include fever with constipation, retention of feces, abdominal pain, and jaundice caused by damp-heat. Chloroplast intergenic spacer *trn*L-F of three genuine species and 10 closely related *Rheum *species were amplified by PCR amplified, and the PCR products were sequenced and aligned. At position 221, the nucleotide for the three genuine species is cytosine while that of the other *Rheum *species is adenine. A pair of specific primers was designed based on this polymorphic site. The three genuine species produce a single 300-bp band after SCAR and the other species give a negative result. Similarly, Chinese Patent 200510031346 was issued for a SCAR sequence for differentiating between *Gastrodia eleata *(*Tianma*) and its adulterant *Lycopus lucidus *(*Zelan*) [52]. With increasing DNA sequences available in the public sequence databases, we have a convenient resource for primer design.

In general, it takes time to identify species-specific SCAR markers, but once found and specific primers designed, authentication of unknown samples becomes simple and routine. SCAR products are specific, as stringent conditions are used when amplifying the DNA. To further improve the resolution and sensitivity of SCAR and DALP, it is important to determine and use highly variable DNA regions and fluorescent primers.

### Patents for high-throughput detection and identification of unknown DNA

DNA identification by PCR-RFLP relies on the presence of restriction cutting sites in the amplified DNA sequence. In the absence of an expected cutting site, which may result from a sequence polymorphism, authentication of a specimen is not possible. Use of DNA hybridization may yield results that are difficult to interpret because hybridization signals may be low. US Patent 7297490 was issued for a novel DNA microarray that provides a high-throughput approach for authenticating Chinese medicinal materials by making use of the variable ribosomal RNA sequences for generating the array [53]. Three medicinal plants, *Ilex asprella*, *Ilex latifolia*, and *Ilex rotunda*, were employed. ITS regions of these samples were first sequenced and compared. Primers were designed for the amplification of the polymorphic ITS-1 and ITS-2 regions. The amplified fragments are spotted onto a nylon membrane. To generate the probe, the whole ITS-1 and ITS-2 sequences are amplified and labeled by DIG-High Prime. After hybridizing the probes to the nylon membrane, signals are detected by DIG Nucleic Acid Detection Kit. When the probe from a particular *Ilex *species is hybridized to the DNA of its own species, a stronger signal is obtained.

### Development after patent application

Patentable advancements have been made in DNA authentication, ranging from the extraction of DNA to high-throughput detection. In particular, inventions that improve the existing protocols for DNA extraction and PCR would have high market value because they can be easily incorporated into existing products. In addition, patented DNA sequences may be employed for designing primers in testing kits or DNA microarrays. Substantial investment will be needed for generating new types of equipment, such as a new DNA extractor. In this case, a patent or a patent application would be especially valuable in terms of securing financial support for developing the technology. After applying for a patent, the inventor would begin to seek for support to further develop the invention. Nevertheless, the application may sometimes be abandoned [54-58], if it is found to have little commercial interest.

## Conclusion

While the demand for DNA authentication is still restricted to endangered, toxic, or high-value medicinal materials, with regulations that require more accurate authentication of herbal materials and an increasing demand for high-quality herbs, the market demand for DNA authentication of medicinal materials will increase, thereby increasing the number of patents for new technologies. Improvements to existing technologies are expected to further advance the field of DNA authentication of herbal materials.

## Competing interests

The authors declare that they have no competing interests.

## Authors' contributions

PCS coordinated the project, revised and edited the manuscript. KLW drafted the manuscript. AWKC and WCW advised on the patent application process and commented on the patents. PPHB advised and revised the manuscript. All authors read and approved the final version of the manuscript.

## Supplementary Material

Additional file 1 **Patentability of some inventions**. This table lists the patent number and reasons for patentability. Click here for file

Additional file 2 **Some abandoned Chinese patent applications**. This table lists the patent application number, area of work and description of the patent and possible reasons for abandoning the application. Click here for file
