# Supplementary material for: Patent applications for using DNA technologies to authenticate medicinal herbal material
Source: Chin Med. 2009 Nov 24;4:21. doi: 10.1186/1749-8546-4-21 (PMC2791102; doi:10.1186/1749-8546-4-21)
Supplement: Additional file 1 — Patentability of some inventions. This table lists the patent number and reasons for patentability. [file 1749-8546-4-21-S1.DOC]

# Patentability of some inventions

| References | Patent number | Reasons for patentability |
| --- | --- | --- |
| [28] | US Patent 6063616 | The examiner did not find any prior art as a basis for rejecting the claims on an apparatus used to break up multiple samples to release the desired substances using electromagnetic micropestles and the claims on methods of extracting molecules using such an apparatus. |
| [29] | US Patent 7214484 | The invention is a nucleic acid extraction kit, which comprises reagent that is free from surfactant/surface-active reagents/detergent/SDS, yielding a greater amount of nucleic acid extract. Because the invention yields better results when compared to its closest prior art (US Patents 6455525 and 6936414) and is novel and non-obvious, a patent is issued. |
| [30] | US Patent 5106966 | This invention is a method to extract DNA using a two-solvent system containing a gel polymer. Although the closest prior art uses silica gel for DNA extraction, it advises against using a two-solvent system. Therefore, the invention is novel and non-obvious. |
| [36] | US Patent 7150980 | This invention uses proline instead of glycerol in primer extension reaction during DNA amplification. Because it is not obvious from the cited references that proline can stabilize and optimize the polymerase activity and is more amenable to primer extension reaction, a patent was issued. |
| [37] | US Patent 6783940 | The invention is incubating a reagent consisting of sorbitol and DMSO of particular concentrations, thereby reducing non-specific PCR amplification. Because the reagent components and concentrations are different from prior art, the invention is patentable. |
| [38] | US Patent 6403341 | The closest prior art segregates magnesium ions from other PCR reagents using grease or wax during PCR. In contrast, this invention sequesters magnesium ions with phosphate ions to form a precipitate, rendering the claimed invention novel and non-obvious. |
| [39] | US Patent 5876977 | Although prior art disclosed the rDNAs extracted from different species of *Panax*, this invention claims the nucleic acid sequences of only the ITS1-5.8S-ITS2 region, and is therefore allowable. Also allowable is a method for determining the source of an herbal material from different species of *Panax* by amplifying the ITS1-5.8S-ITS2 region of extracted rDNA. |
| [40] | Chinese Patent 01102434 | Although prior art disclosed methods for amplifying rDNAs from other herbs, this invention claims the nucleic acid sequences of *Dendrobium* species. This invention is patentable because it provides evidence that such differentiation is possible, but not the methods utilized. |
| [41] | US Patent 6569625 | Although prior art discloses the preparation of *Fritillaria thunbergii* genomic DNA, it does not suggest any differences in the 5S rRNA gene spacer region among the various *Fritillaria* species. Absent evidence of such differences, it is unpredictable whether *Fritillaria* species can be successfully differentiated based on the differences in the 5S rRNA spacer region. This invention is patentable because it provides evidence that such differentiation is possible. |
| [43] | Chinese Patent 03153838 | This invention is directed to the identification of *Equus asinus* using PCR amplification of the cytochrome b with the specific primers, followed by digestion with the restriction enzyme. Although it would have been obvious to apply the step to the identification of animal species, there would be no reasonable expectation of success. Therefore, the claimed invention is novel and non-obvious. |
| [45] | Chinese Patent 01127215 | The invention is directed to the identification of *Eriocheir japonica sinensis* using PCR amplification of polymorphic DNA with the specific primers, followed by digestion with the restriction enzyme *Dra*I. Although it would have been obvious to apply the step to the identification of animal species, the primer pairs are designed by inventors and not reported previously. Therefore, the claimed invention is novel and non-obvious. |
| [46] | Chinese patent 99106135 | This invention is for the identification of *Cordyceps sinensis* using PCR amplification of the 18S rRNA polymorphism with the 18S rRNA-specific primers, NS3 and NS6, followed by digestion with the restriction enzyme *Cfo*I. The cited references identify species using the ITS region. Although it would have been obvious to apply the step to the identification of *C. sinensis*, there would be no reasonable expectation of success. Therefore, the claimed invention is novel and non-obvious. |
| [47] | US Patent 6271003 | This invention is directed to the identification of *Cordyceps sinensis* using PCR amplification of the 18S rRNA polymorphism with the 18S rRNA-specific primers, NS3 and NS6, followed by digestion with the restriction enzyme *Cfo*I. The cited references do not suggest the digestion of PCR products with restriction enzymes. Although it would have been obvious to apply the step to the identification of *C. sinensis*, there would be no reasonable expectation of success. Therefore, the claimed invention is novel and non-obvious. |
| [48] | US Patent 6251606 | This invention utilizes the singularity of the 18S rDNA gene sequence of *Cordyceps* *sinensis* between the NS3/NS6 primer pair as the index for distinguishing *C. sinensis* from other *Cordyceps* species. Because the method of isolating and identifying *C. sinensis* disclosed in the closest prior art, US 5582828, does not suggest amplifying certain regions of the genome, or the specific sequences disclosed in the instant application, the claimed invention is allowed. |
| [49] | US Patent 6803215 | This invention is directed to two methods of identifying an herbal material. One comprises extracting a novel nucleic acid from the material and amplifying a polymorphic region of the nucleic acid. The other comprises extracting a nucleic acid from the material and amplifying a polymorphic region of the nucleic acid with two novel oligonucleotide primers. Because there is a lack of motivation to use the novel nucleic acid or the novel primers, obviousness rejection cannot be established. Hence, a patent is issued. |
| [50] | Chinese Patent 200410016240 | This invention utilizes the specific profiles generated by SCAR for distinguishing wild ginseng from cultivated ginseng and adulterants. The SCAR primers binding to the polymorphic site in the DNA sequences are designed specifically. The primers are novel and therefore the claimed invention is allowed. |
| [51] | Chinese patent 00134133 | This invention utilizes the singularity of the *trn*L-F sequences of *Rheum palmatum*, *R. tanguticum*, or *R. officinale* for distinguishing them from adulterants. The SCAR primers binding to the polymorphic site are designed based on *trn*L-F sequences. The present invention is patentable because it provides evidence that such differentiation is possible. |
| [52] | Chinese Patent 200510031346 | Though the two specific DNA sequences were already disclosed in the reference materials, the usefulness of them for authentication of *Gastrodia eleata* are not proven. The SCAR primers binding to the polymorphic site in the DNA sequences are designed specifically and are novel. Non-obviousness cannot be established, and therefore a patent is issued. |
| [53] | US Patent 7297490 | In contrast to the present invention, prior art did not suggest the technique of collecting different sizes of DNA fragments. There is an additional inventive step of amplifying a variable region of the DNA from a known plant. Therefore the present invention is patentable. |
