# Supplementary material for: Patent applications for using DNA technologies to authenticate medicinal herbal material
Source: Chin Med. 2009 Nov 24;4:21. doi: 10.1186/1749-8546-4-21 (PMC2791102; doi:10.1186/1749-8546-4-21)
Supplement: Additional file 2 — Some abandoned Chinese patent applications. This table lists the patent application number, area of work and description of the patent and possible reasons for abandoning the application. [file 1749-8546-4-21-S2.DOC]

# Some abandoned Chinese patent applications

| References | Patent application number | Area of work | Description of the patent and possible reasons for abandoning the application |
| --- | --- | --- | --- |
| [54] | 03117014 | Amplification of DNA and difficult templates | This application is directed to methods of preserving DNA polymerase activity at 98 °C by adding high concentration of glycerin (4–45%, v/v) to the PCR buffer. Increasing denaturation temperature could overcome the secondary structure of primers and makes PCR amplification of highly GC-rich regions possible. However, such high concentration of glycerin may interfere with the activity of the DNA polymerase. It seems the invention is not practical and the application was abandoned. |
| [55] | 03147806 | Amplification of DNA and difficult templates | This application discloses a method of improving the ramping and stability in the annealing step of PCR by incorporating chemical compounds LC-Red 705 or Digoxigenin (DIG) to the 5’ end of the primer. Nonetheless, labeling the primers with these chemicals is quite expensive and labor-intensive, especially for multiplex-PCR, which always involves more than one pair of primers. Alternative approaches are available on the market, and it seems that the inventors abandoned the application for limited commercial value. |
| [56] | 01104457 | Generation of species-specific fingerprints and sequences | This application utilizes random amplified polymorphic DNA (RAPD) for generating fingerprints to determine whether a powder mixture contains either *Panax ginseng* or *P. quinquefolius*. To generate reproducible RAPD fingerprints, high-quality genomic DNA is required. However, RAPD fingerprints have been shown to vary with the change of DNA template, enzymes, and experimental conditions. The market value is questionable, so the application was abandoned. |
| [57, 58] | 03132205, 200410014259 | High-throughput detection and identification of unknown DNA | Patent application 03132205 discloses a method of designing specific probe for herbal materials by suppression subtraction hybridization (SSH). These species-specific products were cloned and screened by SSH. Clones containing insert were then subjected to PCR using 5’-end chemically modified primers that could be covalently linked onto a glass slide. Patent application 200410014259 discloses a method for the preparation of DNA microarray for five *Dendrobium* species, including using the SSH described in application 03132205. Each *Dendrobium* species has two species-specific DNA fragments spotted onto a glass slide. The mixtures were hybridized to the established DNA microarray. The probes were correctly hybridized to the corresponding target species.  It has been shown that SSH results are not consistent when quality of genomic DNA varies. The market value is questionable, so the application was abandoned. |
